# Tumor-infiltrating granulocytic cells promote cancer cell dissemination

**DOI:** 10.3332/ecancer.2012.241

**Published:** 2012-01-16

**Authors:** B Toh, JP Abastado

**Affiliations:** Singapore Immunology Network, BMSI-A-STAR, 8A Biomedical Grove, #04-06 Immunos, Singapore 138648

## Abstract

Most studies aimed at understanding the link between inflammation and cancer progression have focused on macrophages. In a recent study published in *PLoS Biology*, Toh B *et al* (2011) **Mesenchymal transition and dissemination of cancer cells is driven by myeloid-derived suppressor cells infiltrating the primary tumor** *PLoS Biol* **9** e1001162, we show that a subset of granulocytic cells already known to suppress antitumour immune responses also promotes cancer cell proliferation and metastasis by inducing epithelial to mesenchymal transition. This subset plays a key role in early dissemination of cancer cells throughout the body and may therefore represent a novel target for therapeutic intervention.

## Metastasis as an early event in cancer progression

Metastasis is one of the most important events in cancer progression. Indeed, metastases are usually the cause of death in patients with cancer, not the primary tumor. The prevailing model of metastasis stipulates that cancer cells disseminate to distant organs during the latter stages of disease progression. Over time, cancer cells gain sufficient mutations to allow them to overcome putative barriers to metastasis [1]. However, recent studies from our lab and others have instead shown that cancers cells can disseminate to distant organs at an early stage of primary tumor development, even before the primary tumor can be detected clinically [2–4]. Cancer cells that disseminate early do not immediately give rise to tumors but are instead maintained in a ‘dormant’ state by the immune system, notably by CD8^+^ T cells [4]. Given that cancer cells do not have sufficient time to acquire a metastatic phenotype via random genetic mutations, external stimuli must be responsible for the induction of a metastatic phenotype in these cells. In our recent study published in *PloSBiology* [5], we now show that a specific subset of granulocytic myeloid cells, referred to as granulocytic myeloid-derived suppressor cells (G-MDSC), is responsible for the induction of a metastatic phenotype in the primary tumor, leading to the early dissemination of cancer cells.

MDSC are immature myeloid cells defined by their ability to suppress T cell function. MDSC are broadly made up of two subsets – G-MDSC and monocytic (M)-MDSC, which morphologically resemble granulocytes and monocytes respectively. In addition, G-MDSC are CD11b^+^Gr1^hi^F4/80^−^ as opposed to M-MDSC which are CD11b^+^Gr1^lo^F4/80^+^. Besides these morphological and phenotypic differences, the mediators that determine the immunosuppressive abilities of MDSC also differ between the two subsets. The G-MDSC subset has been shown to be greatly increased in the spleen and blood of tumor-bearing animals and have been shown to be up-regulated in human cancers as well [6]. G-MDSC, though exhibiting morphological and phenotypic similarities to neutrophils, are functionally distinct from neutrophils. The ability to suppress T cell activation and high expression of arginase and reactive oxygen species are some key differences that distinguish G-MDSC from neutrophils [7].

## Accumulation of G-MDSC in primary tumors

This work was carried out in RETAAD mice which spontaneously develop melanoma due to over-expression of the *Rfp-ret* oncogene in melanocytes. We have previously shown that primary tumors develop in the choroid layer of the eye in RETAAD mice, followed by metastasis to distant organs including lungs, lymph nodes, bone marrow and the reproductive tract. Disseminated cancer cells can be detected as early as 3 weeks of age in these animals. This spontaneous tumor model, unlike many transplanted tumor models [8], allows investigators to follow all steps of the metastatic process in animals with a fully competent immune system. We therefore analyzed the immune infiltrate in the tumors of RETAAD mice in order to elucidate the differences between primary and metastatic tumors. We found that there was a preferential accumulation of G-MDSC in the primary tumors of these mice.

**Figure 1:**
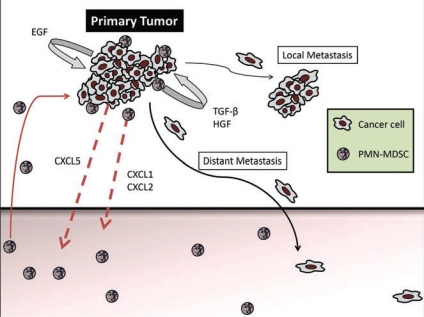
The primary tumor produces CXCL5, which attracts CXCR2-expressing G-MDSC. G-MDSC accumulation is further amplified by autocrine production of CXCL1 and CXCL2. Once located in the primary tumor, G-MDSC induce mesenchymal transition through activation of TGF-β, HGF and EGF pathways, leading to increased cancer cell motility, multi-nodular growth (local metastasis) and dissemination to distant organs.

The accumulation of G-MDSC was due to the expression of CXCR2 ligands, CXCL1, CXCL2 and CXCL5, in the primary tumors but not in the metastases. Indeed, adoptive transfer of either wild-type or CXCR2-knockout bone marrow into RETAAD mice revealed that only wild-type G-MDSC were capable of migrating to the primary tumor, demonstrating an absolute requirement for CXCR2 in mediating G-MDSC accumulation in primary tumors. Furthermore, high expression of CXCL5 by the tumor cells leads us to believe that this is the main factor attracting G-MDSC to the primary tumor. Once present at the tumor site, it is likely that G-MDSC secrete CXCL1 and CXCL2 and attract additional G-MDSC, thus forming a positive feedback loop (figure 1).

## Induction of cancer cell proliferation

To further investigate the role that G-MDSC play in the primary tumor, we developed two schemes to deplete G-MDSC in RETAAD mice; 1) depletion before the onset of the primary tumor at one week of age, and 2) depletion after development of the primary tumor (indicated by exophthalmos at approximately five weeks of age). Interestingly, we found that depletion of G-MDSC resulted in smaller primary tumors with a lower mitotic index. No difference in tumor size could be seen in the metastases, consistent with observed lack of G-MDSC infiltration in these satellite tumors. Increased proliferation of cancer cells could be recapitulated *in vitro* by co-culturing cancer cells with G-MDSC. Although the mechanism of this increased proliferation is still unknown, we determined that secreted factors were critical, because separation of G-MDSC from the cancer cells with a 0.3 micron membrane still resulted in increased proliferation.

## G-MDSC-driven dissemination and mesenchymal transition of cancer cells

Another interesting outcome of depleting G-MDSC was the reduced number of metastases in the skin, lymph nodes and lungs. However, this difference could only be seen if the G-MDSC were depleted before clinical detection of the primary tumor. Depletion of G-MDSC after the age of 5 weeks did not alter the number of metastases. We therefore concluded that G-MDSC were important for cancer cell dissemination, and that cancer cells disseminating before 5 weeks were the main source of metastases. Indeed, primary tumors usually display a multi-nodular morphology, but the frequency of nodules in primary tumors was diminished in the absence of G-MDSC. This finding is also consistent with the observation that metastatic tumors that normally lack G-MDSC do not exhibit a multi-nodular structure. These data led us to hypothesize that G-MDSC are involved in the induction of a motile phenotype in cancer cells that affects both the local site (within the primary tumor) as well as distant tissues (organs outside the eye) by supporting dissemination at an early stage.

Using NBT-II cells (a rat epithelial bladder cancer cell line) as reporter cells *in vitro*, we showed that G-MDSC induced a mesenchymal phenotype that resulted in increased motility of NBT-II cells. This increase was accompanied by down-regulation of E-cadherin on the surface of the NBT-II cells, which is indicative of a mesenchymal-like phenotype. Co-culturing G-MDSC with cancer cells purified from RETAAD tumors, or with a human melanoma cell line, similarly resulted in the down-regulation of E-cadherin *in vitro*. This mesenchymal-like phenotype was also observed *in vivo* in RETAAD tumors, and mesenchymal markers S100A4 and Vimentin were down-regulated in primary tumors depleted of G-MDSC. *In vitro* inhibition of transforming growth factor-β, hepatocyte growth factor and epidermal growth factor, either individually or in combination, was able to partially or completely block the mesenchymal transition. These data indicate that the induction of mesenchymal transition in cancer cells occurs via multiple pathways, although the key factor which initiates this transition process currently remains unknown.

## Phenotypic variability in the primary tumor

G-MDSC stimulated both cancer cell proliferation and mesenchymal transition. However, histopathological examination of the primary tumors revealed that proliferating cancer cells were spread throughout the entire tumor, while those that exhibited a mesenchymal phenotype were located at the edge of the tumor. This distinct distribution might be explained by the fact that G-MDSC induce proliferation through production of soluble factors, while mesenchymal transition seems to require close proximity or even direct contact with cancer cells. Alternatively, we do not rule out the possibility that other immune cells, such as macrophages, may promote mesenchymal transition as well *in vivo*. Thus the locations of the different immune cells throughout the tumor microenvironment might determine the exact nature of the tumor cells in selected areas within the tumor.

The ability of immune cells such as G-MDSC to regulate both the proliferative and metastatic phenotypes of cancer cells compels us to re-visit our current views of cancer progression. With the accumulation of new evidence that supports early cancer cell dissemination, the model of a slowly evolving tumor might not be relevant in this context. The cancer stem cell hypothesis postulates the existence of a defined subset of cancer cells that are able to initiate metastatic tumors. However, could it be that certain cancer cells, due to their location at the edge of the tumor, are simply more susceptible to the effects of the microenvironment, including the secretions of G-MDSC? Could external stimuli such as immune mediators contribute to the ‘stemness’ of these cells? With the emergence of better cancer models such as the RETAAD mouse, it is now possible to observe the tumor as a complex system made up of both cancer cells and tumor stroma. Cancer cells are not isolated but act in concert with various cells in the microenvironment. Understanding these interactions will be key to unlocking this disease.
